# Bis(isonicotinamide-κ*N* ^1^)bis­(4-methyl­benzoato-κ*O*)copper(II) dihydrate

**DOI:** 10.1107/S1600536810028060

**Published:** 2010-07-17

**Authors:** Tuncer Hökelek, Güner Saka, Barış Tercan, Efdal Çimen, Hacali Necefoğlu

**Affiliations:** a Department of Physics, Hacettepe University, 06800 Beytepe, Ankara, Turkey; b Department of Chemistry, Hitit University, 19030 Ulukavak, Çorum, Turkey; c Department of Physics, Karabük University, 78050 Karabük, Turkey; d Department of Chemistry, Kafkas University, 36100 Kars, Turkey

## Abstract

In the centrosymmetric title compound, [Cu(C_8_H_7_O_2_)_2_(C_6_H_6_N_2_O)_2_]·2H_2_O, the Cu^II^ ion is located on a crystallographic inversion center. The asymmetric unit is completed by one 4-methyl­benzoate anion, one isonicotinamide (INA) ligand and one uncoordinated water mol­ecule; all the ligands are monodentate. The two O and the two N atoms around the Cu^II^ ion form a slightly distorted square-planar arrangement. The dihedral angle between the carboxyl­ate group and the attached benzene ring is 13.86 (9)°, while the pyridine and benzene rings are oriented at a dihedral angle of 86.08 (5)°. The uncoordinated water mol­ecules are linked to the INA ligands by O—H⋯O hydrogen bonds. In the crystal structure, inter­molecular O—H⋯O, N—H⋯O and C—H⋯O hydrogen bonds link the mol­ecules into a three-dimensional network.

## Related literature

For niacin, see: Krishnamachari (1974 ▶) and for *N*,*N*-diethyl­nicotinamide, see: Bigoli *et al.* (1972 ▶). For related structures, see: Hökelek *et al.* (1996 ▶, 2009*a* ▶,*b* ▶,*c* ▶); Hökelek & Necefoğlu (1998 ▶); Necefoğlu *et al.* (2010*a* ▶,*b* ▶).
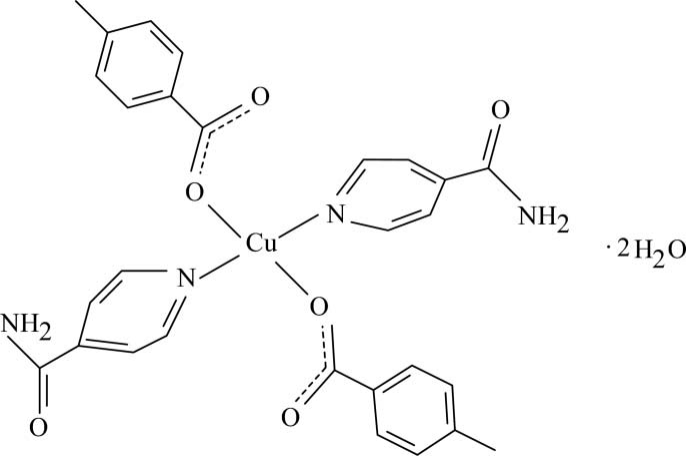


## Experimental

### 

#### Crystal data

[Cu(C_8_H_7_O_2_)_2_(C_6_H_6_N_2_O)_2_]·2H_2_O
*M* *_r_* = 614.11
Monoclinic, 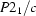

*a* = 5.7138 (2) Å
*b* = 18.9948 (4) Å
*c* = 11.9671 (3) Å
β = 95.906 (3)°
*V* = 1291.92 (6) Å^3^
*Z* = 2
Mo *K*α radiation
μ = 0.91 mm^−1^
*T* = 100 K
0.29 × 0.27 × 0.25 mm

#### Data collection

Bruker Kappa APEXII CCD area-detector diffractometer
Absorption correction: multi-scan (*SADABS*; Bruker, 2005 ▶) *T*_min_ = 0.763, *T*_max_ = 0.959
11754 measured reflections
3199 independent reflections
2637 reflections with *I* > 2σ(*I*)
*R*_int_ = 0.026

#### Refinement

*R*[*F* ^2^ > 2σ(*F* ^2^)] = 0.035
*wR*(*F* ^2^) = 0.080
*S* = 1.08
3199 reflections
204 parameters
H atoms treated by a mixture of independent and constrained refinement
Δρ_max_ = 0.63 e Å^−3^
Δρ_min_ = −0.51 e Å^−3^

### 

Data collection: *APEX2* (Bruker, 2007 ▶); cell refinement: *SAINT* (Bruker, 2007 ▶); data reduction: *SAINT*; program(s) used to solve structure: *SHELXS97* (Sheldrick, 2008 ▶); program(s) used to refine structure: *SHELXL97* (Sheldrick, 2008 ▶); molecular graphics: *ORTEP-3 for Windows* (Farrugia, 1997 ▶); software used to prepare material for publication: *WinGX* (Farrugia, 1999 ▶).

## Supplementary Material

Crystal structure: contains datablocks I, global. DOI: 10.1107/S1600536810028060/su2197sup1.cif

Structure factors: contains datablocks I. DOI: 10.1107/S1600536810028060/su2197Isup2.hkl

Additional supplementary materials:  crystallographic information; 3D view; checkCIF report

## Figures and Tables

**Table 1:** Hydrogen-bond geometry (Å, °)

*D*—H⋯*A*	*D*—H	H⋯*A*	*D*⋯*A*	*D*—H⋯*A*
N2—H21⋯O1^i^	0.82 (2)	2.46 (2)	3.283 (2)	177 (2)
N2—H22⋯O4^ii^	0.86 (2)	2.14 (2)	2.983 (2)	171 (2)
O4—H41⋯O3	0.77 (3)	2.10 (3)	2.866 (2)	178 (3)
O4—H42⋯O1^iii^	0.78 (3)	2.04 (3)	2.813 (2)	173 (3)
C3—H3⋯O3^iv^	0.93	2.48	3.324 (2)	151
C10—H10⋯O1^iii^	0.93	2.50	3.242 (2)	137
C12—H12⋯O4^ii^	0.93	2.34	3.253 (2)	169

Symmetry codes: (i) 
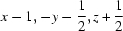
; (ii) 
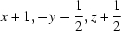
; (iii) 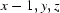; (iv) 
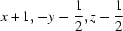
.

**Fig. 1.**
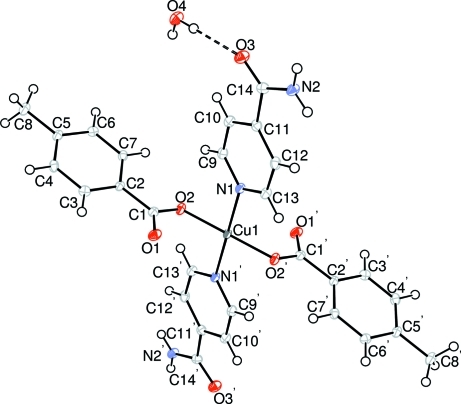
The molecular structure of the title compound with the atom-numbering scheme. Displacement ellipsoids are drawn at the 50% probability level. Primed atoms are generated by the symmetry operator: (') -x, -y, -z. Only one of the crystal water molecules is shown [dashed line indicates the O-H···O hydrogen-bond].

## supplementary crystallographic information

### Comment

As a part of our ongoing investigation on transition metal complexes of nicotinamide (NA), one form of niacin (Krishnamachari, 1974), and/or the nicotinic acid derivative *N*,*N*-diethylnicotinamide (DENA), an important respiratory stimulant (Bigoli *et al.*, 1972), the title compound was synthesized and its crystal structure is reported herein.

The title compound is a mononuclear complex, where the Cu^II^ ion is located on a crystallographic inversion center (Fig. 1). The asymmetric unit contains one 4-methylbenzoate (PMB) anion, one isonicotinamide (INA) ligand and one uncoordinated water molecule, and all the ligands are coordinated in a monodentate manner. The crystal structures of some NA and/or DENA complexes of Cu^II^, Co^II^, Ni^II^, Mn^II^ and Zn^II^ ions have been reported on recently (Hökelek *et al.*, 1996; Necefoğlu *et al.*, 2010**a*,b*; Hökelek & Necefoğlu, 1998; Hökelek *et al.*, 2009**a*,*b*,c*. In the copper(II) complex, *trans*-Bis(benzoato-O,O')bis(N,N-diethylnicotinamide-N1)copper(II) [Hökelek *et al.*, 1996], the two benzoate ions are coordinated to the Cu atom as bidentate ligands, while in the other structures all the ligands are coordinated in a monodentate manner.

The two O atoms (O2, and the symmetry-related atom, O2') and the two N atoms (N1, and the symmetry-related atom, N1') around the Cu^II^ ion form a slightly distorted square-planar arrangement (Fig. 1). The Cu1—O2 bond length is 1.9192 (13) Å, and the Cu1—N1 bond length is 2.0562 (15) Å.The near equality of the C1—O1 [1.249 (2) Å] and C1—O2 [1.287 (2) Å] bonds in the carboxylate group indicates a delocalized bonding arrangement, rather than localized single and double bonds. The Cu^II^ ion is displaced out of the least-squares plane of the carboxylate group (O1/C1/O2) by 0.3593 (1) Å. The dihedral angle between the planar carboxylate group and the benzene ring A (= C2—C7) is 13.86 (9)°, while that between rings A and B (= N1/C9—C13) is 86.08 (5)°. The uncoordinated water molecules are linked to the INA groups by O-H···O hydrogen bonds (Table 1 and Fig. 1).

In the crystal structure intermolecular O—H···O, N—H···O and C—H···O hydrogen bonds (Table 1) link the molecules to form a three-dimensional network.

### Experimental

The title compound was prepared by the reaction of CuSO_4_.5H_2_O (1.25 g, 5 mmol) in H_2_O (50 ml) and isonicotinamide (2.44 g, 20 mmol) in H_2_O (15 ml) with sodium 4-methylbenzoate (1.58 g, 10 mmol) in H_2_O (500 ml). The precipitated green mass was set aside in solution at ambient temperature. After three weeks it had transformed into purple crystals. These were separated off by filtration and dried at room temperature.

### Refinement

Atoms H21, H22 (for NH_2_) and H41, H42 (for H_2_O) were located in a difference Fourier map and were freely refined: N-H = 0.82 (2) - 0.86 (2) Å; O-H = 0.77 (3) - 0.78 (3) Å. The remaining H atoms were positioned geometrically with C—H = 0.93 and 0.96 Å for aromatic and methyl H-atoms, respectively, and constrained to ride on their parent atoms, with U_iso_(H) = k × U_eq_(C), where k = 1.5 for methyl H-atoms and k = 1.2 for aromatic H-atoms.

### Crystal data

**Table:**

[Cu(C_8_H_7_O_2_)_2_(C_6_H_6_N_2_O)_2_]·2H_2_O	*F*(000) = 638
*M**_r_* = 614.11	*D*_x_ = 1.579 Mg m^−^^3^
Monoclinic, *P*2_1_/*c*	Mo *K*α radiation, λ = 0.71073 Å
Hall symbol: -P 2ybc	Cell parameters from 5200 reflections
*a* = 5.7138 (2) Å	θ = 2.7–28.2°
*b* = 18.9948 (4) Å	µ = 0.91 mm^−^^1^
*c* = 11.9671 (3) Å	*T* = 100 K
β = 95.906 (3)°	Block, violet
*V* = 1291.92 (6) Å^3^	0.29 × 0.27 × 0.25 mm
*Z* = 2	

### Data collection

**Table:**

Bruker Kappa APEXII CCD area-detector diffractometer	3199 independent reflections
Radiation source: fine-focus sealed tube	2637 reflections with *I* > 2σ(*I*)
graphite	*R*_int_ = 0.026
φ and ω scans	θ_max_ = 28.4°, θ_min_ = 2.0°
Absorption correction: multi-scan (*SADABS*; Bruker, 2005)	*h* = −7→7
*T*_min_ = 0.763, *T*_max_ = 0.959	*k* = −20→25
11754 measured reflections	*l* = −16→14

### Refinement

**Table:**

Refinement on *F*^2^	Primary atom site location: structure-invariant direct methods
Least-squares matrix: full	Secondary atom site location: difference Fourier map
*R*[*F*^2^ > 2σ(*F*^2^)] = 0.035	Hydrogen site location: inferred from neighbouring sites
*wR*(*F*^2^) = 0.080	H atoms treated by a mixture of independent and constrained refinement
*S* = 1.08	*w* = 1/[σ^2^(*F*_o_^2^) + (0.0303*P*)^2^ + 1.1079*P*] where *P* = (*F*_o_^2^ + 2*F*_c_^2^)/3
3199 reflections	(Δ/σ)_max_ < 0.001
204 parameters	Δρ_max_ = 0.63 e Å^−^^3^
0 restraints	Δρ_min_ = −0.51 e Å^−^^3^

### Special details

**Table:**

Geometry. All esds (except the esd in the dihedral angle between two l.s. planes) are estimated using the full covariance matrix. The cell esds are taken into account individually in the estimation of esds in distances, angles and torsion angles; correlations between esds in cell parameters are only used when they are defined by crystal symmetry. An approximate (isotropic) treatment of cell esds is used for estimating esds involving l.s. planes.
Refinement. Refinement of F^2^ against ALL reflections. The weighted R-factor wR and goodness of fit S are based on F^2^, conventional R-factors R are based on F, with F set to zero for negative F^2^. The threshold expression of F^2^ > 2sigma(F^2^) is used only for calculating R-factors(gt) etc. and is not relevant to the choice of reflections for refinement. R-factors based on F^2^ are statistically about twice as large as those based on F, and R- factors based on ALL data will be even larger.

### Fractional atomic coordinates and isotropic or equivalent isotropic displacement parameters (Å^2^)

**Table:**

	*x*	*y*	*z*	*U*_iso_*/*U*_eq_	
Cu1	0.0000	0.0000	0.0000	0.01074 (9)	
O1	0.0382 (2)	−0.09651 (7)	−0.17689 (11)	0.0173 (3)	
O2	−0.1745 (2)	−0.00164 (6)	−0.14584 (11)	0.0140 (3)	
O3	−0.8594 (2)	−0.25069 (7)	0.09449 (12)	0.0191 (3)	
O4	−1.1555 (3)	−0.22382 (8)	−0.10917 (14)	0.0184 (3)	
H41	−1.077 (5)	−0.2318 (14)	−0.055 (2)	0.027 (7)*	
H42	−1.097 (5)	−0.1903 (14)	−0.132 (2)	0.028 (7)*	
N1	−0.2249 (3)	−0.07406 (8)	0.05451 (13)	0.0124 (3)	
N2	−0.6168 (3)	−0.27723 (9)	0.24930 (14)	0.0158 (3)	
H21	−0.707 (4)	−0.3082 (12)	0.2657 (19)	0.016 (6)*	
H22	−0.487 (4)	−0.2719 (10)	0.2909 (18)	0.007 (5)*	
C1	−0.1300 (3)	−0.05595 (9)	−0.20439 (15)	0.0134 (4)	
C2	−0.2973 (3)	−0.06975 (9)	−0.30646 (15)	0.0112 (3)	
C3	−0.2437 (3)	−0.11966 (9)	−0.38569 (16)	0.0144 (4)	
H3	−0.1009	−0.1435	−0.3763	0.017*	
C4	−0.4027 (3)	−0.13357 (9)	−0.47810 (16)	0.0144 (4)	
H4	−0.3645	−0.1665	−0.5308	0.017*	
C5	−0.6202 (3)	−0.09904 (9)	−0.49401 (15)	0.0132 (4)	
C6	−0.6723 (3)	−0.04968 (9)	−0.41381 (16)	0.0136 (4)	
H6	−0.8162	−0.0264	−0.4225	0.016*	
C7	−0.5137 (3)	−0.03481 (9)	−0.32179 (16)	0.0126 (4)	
H7	−0.5510	−0.0013	−0.2697	0.015*	
C8	−0.7908 (3)	−0.11485 (10)	−0.59515 (16)	0.0175 (4)	
H8A	−0.8165	−0.1647	−0.6006	0.026*	
H8B	−0.7274	−0.0984	−0.6616	0.026*	
H8C	−0.9374	−0.0915	−0.5878	0.026*	
C9	−0.4391 (3)	−0.08763 (10)	0.00027 (16)	0.0143 (4)	
H9	−0.4908	−0.0604	−0.0621	0.017*	
C10	−0.5851 (3)	−0.14000 (9)	0.03306 (16)	0.0149 (4)	
H10	−0.7323	−0.1471	−0.0062	0.018*	
C11	−0.5111 (3)	−0.18220 (9)	0.12538 (16)	0.0132 (4)	
C12	−0.2892 (3)	−0.16896 (9)	0.18202 (16)	0.0149 (4)	
H12	−0.2327	−0.1958	0.2440	0.018*	
C13	−0.1551 (3)	−0.11484 (10)	0.14368 (16)	0.0150 (4)	
H13	−0.0079	−0.1062	0.1820	0.018*	
C14	−0.6762 (3)	−0.24014 (9)	0.15535 (16)	0.0145 (4)	

### Atomic displacement parameters (Å^2^)

**Table:**

	*U*^11^	*U*^22^	*U*^33^	*U*^12^	*U*^13^	*U*^23^
Cu1	0.01320 (16)	0.01035 (15)	0.00829 (16)	−0.00275 (12)	−0.00076 (11)	−0.00013 (12)
O1	0.0140 (7)	0.0194 (7)	0.0172 (7)	0.0006 (5)	−0.0041 (5)	0.0042 (5)
O2	0.0181 (6)	0.0127 (6)	0.0103 (6)	−0.0040 (5)	−0.0020 (5)	0.0005 (5)
O3	0.0180 (7)	0.0167 (6)	0.0217 (8)	−0.0030 (5)	−0.0025 (6)	0.0026 (6)
O4	0.0205 (8)	0.0147 (7)	0.0189 (8)	−0.0022 (6)	−0.0036 (6)	0.0018 (6)
N1	0.0131 (8)	0.0128 (7)	0.0110 (8)	−0.0015 (6)	−0.0007 (6)	−0.0005 (6)
N2	0.0154 (9)	0.0143 (8)	0.0168 (9)	−0.0034 (6)	−0.0019 (7)	0.0021 (6)
C1	0.0144 (9)	0.0138 (8)	0.0118 (9)	−0.0058 (7)	0.0011 (7)	0.0037 (7)
C2	0.0129 (9)	0.0102 (8)	0.0102 (9)	−0.0029 (6)	−0.0004 (7)	0.0014 (6)
C3	0.0139 (9)	0.0128 (8)	0.0164 (10)	0.0011 (7)	0.0013 (7)	0.0016 (7)
C4	0.0195 (9)	0.0109 (8)	0.0131 (9)	−0.0004 (7)	0.0024 (7)	−0.0036 (7)
C5	0.0163 (9)	0.0117 (8)	0.0110 (9)	−0.0045 (7)	−0.0010 (7)	0.0016 (7)
C6	0.0130 (9)	0.0127 (8)	0.0149 (10)	0.0003 (6)	0.0003 (7)	0.0019 (7)
C7	0.0155 (9)	0.0106 (8)	0.0117 (9)	−0.0016 (6)	0.0014 (7)	−0.0011 (7)
C8	0.0205 (10)	0.0174 (9)	0.0136 (10)	−0.0034 (7)	−0.0032 (8)	−0.0003 (7)
C9	0.0161 (9)	0.0142 (9)	0.0124 (9)	0.0014 (6)	0.0000 (7)	−0.0001 (7)
C10	0.0148 (9)	0.0147 (9)	0.0149 (10)	0.0004 (7)	−0.0004 (7)	−0.0015 (7)
C11	0.0152 (9)	0.0110 (8)	0.0137 (10)	0.0005 (6)	0.0028 (7)	−0.0014 (7)
C12	0.0200 (10)	0.0130 (8)	0.0116 (9)	−0.0013 (7)	0.0012 (7)	0.0014 (7)
C13	0.0149 (9)	0.0164 (9)	0.0131 (9)	−0.0001 (7)	−0.0013 (7)	0.0001 (7)
C14	0.0133 (9)	0.0120 (8)	0.0183 (10)	0.0011 (7)	0.0027 (7)	−0.0012 (7)

### Geometric parameters (Å, °)

**Table:**

Cu1—O2	1.9192 (13)	C4—H4	0.9300
Cu1—O2^i^	1.9192 (13)	C5—C4	1.401 (3)
Cu1—N1	2.0562 (15)	C5—C6	1.396 (3)
Cu1—N1^i^	2.0562 (15)	C5—C8	1.504 (3)
O1—C1	1.249 (2)	C6—H6	0.9300
O2—C1	1.287 (2)	C7—C6	1.382 (3)
O3—C14	1.228 (2)	C7—H7	0.9300
O4—H41	0.77 (3)	C8—H8A	0.9600
O4—H42	0.78 (3)	C8—H8B	0.9600
N1—C9	1.350 (2)	C8—H8C	0.9600
N1—C13	1.345 (2)	C9—H9	0.9300
N2—C14	1.341 (2)	C10—C9	1.381 (3)
N2—H21	0.82 (2)	C10—C11	1.395 (3)
N2—H22	0.86 (2)	C10—H10	0.9300
C2—C1	1.495 (3)	C11—C12	1.398 (3)
C2—C3	1.397 (2)	C11—C14	1.516 (2)
C2—C7	1.398 (3)	C12—C13	1.388 (2)
C3—C4	1.383 (3)	C12—H12	0.9300
C3—H3	0.9300	C13—H13	0.9300
			
O2^i^—Cu1—O2	180.00 (4)	C7—C6—C5	121.11 (17)
O2—Cu1—N1	89.67 (6)	C7—C6—H6	119.4
O2^i^—Cu1—N1	90.33 (6)	C2—C7—H7	119.8
O2—Cu1—N1^i^	90.33 (6)	C6—C7—C2	120.40 (17)
O2^i^—Cu1—N1^i^	89.67 (6)	C6—C7—H7	119.8
N1—Cu1—N1^i^	180.00 (10)	C5—C8—H8A	109.5
C1—O2—Cu1	113.26 (12)	C5—C8—H8B	109.5
H42—O4—H41	103 (3)	C5—C8—H8C	109.5
C9—N1—Cu1	122.91 (12)	H8A—C8—H8B	109.5
C13—N1—Cu1	120.10 (12)	H8A—C8—H8C	109.5
C13—N1—C9	116.85 (16)	H8B—C8—H8C	109.5
C14—N2—H21	117.9 (16)	N1—C9—C10	123.05 (17)
C14—N2—H22	123.8 (14)	N1—C9—H9	118.5
H22—N2—H21	118 (2)	C10—C9—H9	118.5
O1—C1—O2	122.72 (18)	C9—C10—C11	119.75 (17)
O1—C1—C2	121.14 (17)	C9—C10—H10	120.1
O2—C1—C2	116.10 (16)	C11—C10—H10	120.1
C3—C2—C1	120.47 (16)	C10—C11—C12	117.82 (17)
C3—C2—C7	119.07 (17)	C10—C11—C14	117.32 (16)
C7—C2—C1	120.42 (16)	C12—C11—C14	124.85 (17)
C2—C3—H3	120.0	C11—C12—H12	120.7
C4—C3—C2	120.03 (17)	C13—C12—C11	118.51 (17)
C4—C3—H3	120.0	C13—C12—H12	120.7
C3—C4—C5	121.34 (17)	N1—C13—C12	124.04 (17)
C3—C4—H4	119.3	N1—C13—H13	118.0
C5—C4—H4	119.3	C12—C13—H13	118.0
C4—C5—C8	120.56 (16)	O3—C14—N2	122.56 (17)
C6—C5—C4	118.05 (17)	O3—C14—C11	119.40 (17)
C6—C5—C8	121.39 (17)	N2—C14—C11	118.03 (17)
C5—C6—H6	119.4		
			
O2^i^—Cu1—N1—C9	161.86 (14)	C1—C2—C7—C6	−177.34 (16)
O2—Cu1—N1—C9	−18.14 (14)	C3—C2—C7—C6	0.2 (3)
O2^i^—Cu1—N1—C13	−22.59 (14)	C2—C3—C4—C5	−0.7 (3)
O2—Cu1—N1—C13	157.41 (14)	C6—C5—C4—C3	0.2 (3)
N1—Cu1—O2—C1	−76.89 (12)	C8—C5—C4—C3	179.96 (17)
N1^i^—Cu1—O2—C1	103.11 (12)	C4—C5—C6—C7	0.5 (3)
Cu1—O2—C1—O1	−11.8 (2)	C8—C5—C6—C7	−179.23 (16)
Cu1—O2—C1—C2	166.15 (11)	C2—C7—C6—C5	−0.7 (3)
Cu1—N1—C9—C10	176.28 (14)	C11—C10—C9—N1	−0.8 (3)
C13—N1—C9—C10	0.6 (3)	C9—C10—C11—C12	0.5 (3)
Cu1—N1—C13—C12	−175.86 (14)	C9—C10—C11—C14	−178.32 (16)
C9—N1—C13—C12	0.0 (3)	C10—C11—C12—C13	0.0 (3)
C3—C2—C1—O1	−13.1 (3)	C14—C11—C12—C13	178.73 (17)
C7—C2—C1—O1	164.51 (16)	C10—C11—C14—O3	4.5 (2)
C3—C2—C1—O2	169.00 (16)	C10—C11—C14—N2	−174.41 (17)
C7—C2—C1—O2	−13.4 (2)	C12—C11—C14—O3	−174.24 (18)
C7—C2—C3—C4	0.5 (3)	C12—C11—C14—N2	6.9 (3)
C1—C2—C3—C4	178.05 (16)	C11—C12—C13—N1	−0.3 (3)

Symmetry codes: (i) −*x*, −*y*, −*z*.

### Hydrogen-bond geometry (Å, °)

**Table:**

*D*—H···*A*	*D*—H	H···*A*	*D*···*A*	*D*—H···*A*
N2—H21···O1^ii^	0.82 (2)	2.46 (2)	3.283 (2)	177 (2)
N2—H22···O4^iii^	0.86 (2)	2.14 (2)	2.983 (2)	171 (2)
O4—H41···O3	0.77 (3)	2.10 (3)	2.866 (2)	178 (3)
O4—H42···O1^iv^	0.78 (3)	2.04 (3)	2.813 (2)	173 (3)
C3—H3···O3^v^	0.93	2.48	3.324 (2)	151
C10—H10···O1^iv^	0.93	2.50	3.242 (2)	137
C12—H12···O4^iii^	0.93	2.34	3.253 (2)	169

Symmetry codes: (ii) *x*−1, −*y*−1/2, *z*+1/2; (iii) *x*+1, −*y*−1/2, *z*+1/2; (iv) *x*−1, *y*, *z*; (v) *x*+1, −*y*−1/2, *z*−1/2.
